# Comparison of pain, functional and psychological trajectories between total and unicompartmental knee arthroplasties: secondary analysis of a 6-month prospective observational study

**DOI:** 10.1007/s00402-024-05710-x

**Published:** 2024-12-12

**Authors:** Marc Terradas-Monllor, Sandra Rierola-Fochs, Jose Antonio Merchan-Baeza, Carles Parés-Martinez, Cristina Font-Jutglà, José A. Hernández-Hermoso, Mirari Ochandorena-Acha

**Affiliations:** 1 Research Group on Methodology, Methods, Models and Outcomes of Health and Social Sciences (M3O), Faculty of Health Sciences and Welfare, Centre for Health and Social Care Research (CESS), University of Vic-Central University of Catalonia (UVic-UCC), Vic, Spain; 2 Institute for Research and Innovation in Life Sciences and Health in Central Catalonia (IRIS-CC), Vic, Spain; 3 Pain Medicine Section, Anaesthesiology Department, Hospital Clinic de Barcelona, Barcelona, Spain; 4 Department of Orthopedic Surgery and Traumatology Hospital, Universitari Germans Trias i Pujol, Carretera Canyet s/n, Badalona, 08916 Barcelona, Spain; 5 Department of Surgery. Faculty of Medicine, Universitat Autònoma de Barcelona, Campus UAB, 08913 Bellaterra, Spain

**Keywords:** Total knee arthroplasty, Unicompartmental knee arthroplasty, Knee replacement, Postsurgical pain

## Abstract

**Introduction:**

Unicompartmental knee arthroplasty (UKA) treats osteoarthritis in one knee compartment, while total knee arthroplasty (TKA) addresses all compartments. The debate focuses on UKA's advantages of quicker recovery and fewer complications versus TKA's lower long-term revision rates, emphasizing the need for thorough outcome evaluations. The aim of the present study is to describe and compare the pain, functional and psychological trajectories during a 6-month postoperative rehabilitation period between total and unicompartmental knee arthroplasties.

**Materials and Methods:**

115 participants who had undergone either TKA or UKA were recruited. Outcome measurements were performed at 1, 4, 12 and 24 weeks post-surgery. Measurements included pain intensity (Visual Analog Scale), range of motion, walking speed (4 m walking test), physical performance (30-s chair stand test), health functioning (Western Ontario and McMaster Universities Osteoarthritis Index), pain catastrophizing (pain catastrophizing scale), fear of movement (Tampa Scale of Kinesiophobia), anxiety and depression (Hospital Anxiety and Depression Scale). A mixed-effects model was used to estimate the influence of type of surgery (either unicompartmental or total knee arthroplasty) to pain, function, and psychological trajectories.

**Results:**

Both TKA and UKA groups showed significant improvements across the six-month rehabilitation period except for anxiety symptoms in the TKA group, and fear of movement and depression in the UKA group. Between group analysis revealed that in the acute phase UKA patients showed improved range of motion and TKA patients displayed faster walking speed but higher fear of movement. Overall, the type of surgery does not significantly influence the overall rehabilitation pain, functional and psychological trajectories.

**Conclusions:**

Despite differences in the acute phase, there are no differences in pain, functional and psychological trajectories throughout the six-month rehabilitation period. These results should be acknowledged to better inform patients and to improve patient education during the perioperative period.

**Trial registration number:**

NCT03378440 (2017-12-18), retrospectively registered.

**Level of evidence:**

Level II.

## Introduction

Knee osteoarthritis (OA) stands as the most prevalent joint disease in older adults(1,2). Moreover, the likelihood of developing symptomatic knee OA during one’s lifetime is approximately 40% for men and 47% for women, with women experiencing higher prevalence and severity(3). While pain is the primary symptom, joint stiffness (often brief), deformity, crepitus, weakness, or instability may also be present(4). Furthermore, knee OA consistently diminishes patients' capacity to perform daily activities and work, thereby imposing a significant economic burden on society(5). Unicompartmental knee arthroplasty (UKA) is a surgical solution tailored for treating OA within one knee compartment, predominantly the anteromedial compartment, in contrast with total knee arthroplasty (TKA), which addresses OA across all three compartments(6). Since its introduction in the 1970s(7),proponents highlighted its potential to mirror natural knee kinematics, reduce perioperative morbidity and blood loss, and enable quicker rehabilitation compared to traditional methods(8). However, outcomes revealed higher revision rates, with a substantial proportion requiring conversion to TKA(9). Despite improvements in implant design, surgical techniques, and expanded indications, it has been demonstrated that the best way to minimize the revision rate is for surgeons to perform UKA for at least 20% of their knee arthroplasties(10).

Within the landscape of knee arthroplasty, understanding the trajectories of pain, functional outcomes, and psychological profiles remains limited yet paramount(11). These aspects are particularly vital within established protocols like the Fast-Track protocol(12). Notably, dissatisfaction with surgical outcomes among 20% of patients underscores the necessity for focused research and development efforts(11). Additionally, the aging demographic increasingly grapples with functionally limiting knee OA, driving a surge in TKA surgeries to uphold quality of life(11).

The burgeoning utilization rates of knee replacement globally are multifaceted, driven by various factors. Economic evaluations suggest that while TKA generally proves cost-effective, significant heterogeneity in costs and benefits across patient subgroups persists(11). The debate over the preferred treatment option between UKA and TKA continues, with evidence favoring UKA for its reduced complications, mortality rates, and faster recovery(13). However, the decision-making process should be collaborative, considering the limited comparative data from randomized controlled trials and methodological variations across studies. Notably, UKA demonstrates short-term benefits like reduced hospital stays and better functional outcomes but carries a higher long-term risk of revision compared to TKA, albeit with an overall lower reoperation rate(13). Thus, comprehensive outcome assessments are imperative, especially amidst the absence of consensus on the optimal treatment choice.

The aim of the present study is to describe and compare the pain, functional and psychological trajectories during a 6-month postoperative rehabilitation period between total and unicompartmental knee arthroplasties.

## Methods

This research adhered to the guidelines of the Strengthening the Reporting of Observational Studies in Epidemiology (STROBE) statement (14) and complied with the Declaration of Helsinki principles. The study protocol was reviewed and approved by the Research Ethics Committee of the University of Vic – Central University of Catalonia (approval number: 59/2018). The current study represents a secondary analysis of a protocol previously registered at ClinicalTrials.gov (Identifier: NCT03378440). All participants provided their consent to participate by signing an informed consent form.

### Study design and setting

A prospective cohort study with a six-month duration was conducted from December 2018 to January 2020. A total of 115 patients were consecutively recruited from December 2018 through May 2020 via a domiciliary rehabilitation service in Barcelona, Spain, employing a non-random selection strategy.

### Participants

Eligibility for recruitment was considered for patients who had undergone either total or unicompartmental knee arthroplasty (KA) due to primary osteoarthritis. Exclusion criteria, evaluated by a general practitioner, included: (1) patients with revision surgery, (2) surgery performed for secondary osteoarthritis, (3) inability to read or speak Spanish, (4) diagnosis of inflammatory arthritis, and (5) enrollment in domiciliary physical therapy more than one-week post-surgery. The latter criterion was due to delays in documentation processing or logistical issues preventing the patient from starting rehabilitation in time for the baseline assessment (one week post-surgery).

All surgeries were performed by an experienced surgical team that conducts over 20 UKAs per year, accounting more than 20% of surgeon’s knee arthroplasty volume. All participants received a cemented posterior stabilized TKA and a cemented fix bearing UKA, followed by the same postoperative protocol.

### Outcome measurements

Outcome measurements were performed at patients' homes 1, 4, 12, and 24 weeks post-surgery by three physiotherapists, with the same physiotherapist assessing the same participant at each follow-up.

#### Demographic and health characteristics

At baseline, demographic and health-related data were collected, encompassing age, sex, body mass index (BMI), comorbidities (evaluated using the Charlson comorbidity index) (15), alcohol and smoking habits, and educational level.

#### Pain trajectory variables

Pain intensity was quantified using a 100-mm visual analog scale (VAS), with endpoints defined as 0 (no pain) and 100 (the worst imaginable pain) (16). Patients reported their average pain experienced during the previous week under conditions of rest, walking, and knee bending.

#### Functional trajectory variables

Knee flexion movement's active range of motion (ROM) was measured using a universal goniometer, a tool validated for its accuracy and reliability in knee ROM assessments (17). Walking speed was evaluated using the 4 m walking test (4mWT), conducted thrice to calculate an average score, with allowances for assistive devices (18). Physical performance was gauged through the 30-s chair stand test (30s CST), requiring participants to repeatedly sit and stand from a standard chair (19). The Western Ontario and McMaster Universities Osteoarthritis Index (WOMAC), in its Spanish version, assessed health functioning across three domains: pain, stiffness, and physical function (20).

#### Psychological trajectory variables

The Spanish versions of the Pain Catastrophizing Scale (PCS) and the Tampa Scale of Kinesiophobia (TSK-11) were used to evaluate pain catastrophizing and fear of movement, respectively (21,22). Anxiety and depressive symptoms were assessed with the Hospital Anxiety and Depression Scale (HADS), which includes separate subscales for anxiety and depression (23).

### Biases

One of the physical therapists engaged in data collection also participated in the statistical analysis and was aware of the surgery types, potentially introducing detection bias. To mitigate biases associated with automated response patterns due to fatigue or recall of previously answered questions, self-administered questionnaires were distributed in a random sequence. Additionally, to ensure uniformity in the physical measurements, all participating physiotherapists convened prior to the commencement of the study to agree upon standardized criteria. Since this study is a secondary analysis of an existing dataset, it is also limited by the original study design, which may introduce selection bias and impact the validity of the findings.

### Study size

A sample size calculation was not conducted for this study, as it constitutes a secondary analysis of data from a prior observational study that was designed with a distinctly different hypothesis and objective(24). This absence of an initial sample size determination should be acknowledged as a limitation, as it may result in the current study being underpowered.

### Data analysis

Continuous variables were characterized using means and standard deviations, while categorical variables were delineated through raw frequencies and percentages. The normality of continuous variables was assessed using the Kolmogorov–Smirnov test. To compare baseline characteristics of the sample, both the nonparametric Mann–Whitney U test for continuous variables and χ^2^ tests for categorical variables were employed.

Initially, separate models were developed to describe the temporal evolution of variables related to pain, function, and psychological trajectories. A mixed-effects model was utilized to estimate these trajectories, incorporating a fixed effect for the intervals between measurement points and a random effect to account for individual variability. This model served as a baseline for comparison when integrating additional covariates. Additionally, a between-group analyses at each measurement point was performed via the Mann–Whitney U test. Subsequently, covariates such as sex, body mass index, comorbidities, alcohol or smoking habits, and education level were included in the models to control for their effects if found significant. Finally, the type of surgery (either unicompartmental or total knee arthroplasty) was incorporated as a covariate in all models to evaluate its distinct impact.

For all analyses, P values less than 0.05 were deemed to indicate statistical significance. All statistical analyses were conducted using IBM SPSS, version 29 (IBM, Chicago, IL, USA).

## Results

**Fig. 1:**
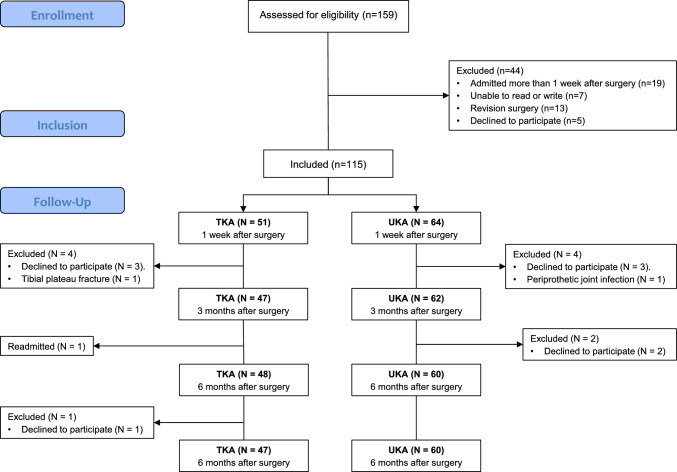
Flow diagram of participants in the study

A total of 159 patients were evaluated for eligibility, of which 115 met the inclusion criteria and consented to participate in the study. Among these participants, 51 underwent total knee arthroplasty, while 64 received unicompartmental knee arthroplasty. Detailed distribution of the study population is depicted in Fig. 1. Baseline patient characteristics of the overall and segregated cohort are summarized in Table 1. No differences between groups were observed in any variables.

**Table 1:** Sample characteristics at baseline

Variables	Total sample (N = 115)	TKA subjects (N = 51)	UKA subjects (N = 64)	p-value
Age (years of age)	70.45 (7.72)	70.92 (7.65)	70.07 (7.82)	0.826
Sex (female), n (%)	76 (66.10)	38 (74.50)	38 (59.40)	0.089
Body mass index (BMI)	30.84 (5.18)	31.04 (5.11)	30.67 (5.26)	0.724
Charlson Comorbidity Index (CCI)	3.02 (1.12)	3.08 (1.11)	2.97 (1.14)	0.687
*Alcohol habit, n (%)*				0.445
Never	49 (42.60)	21 (41.20)	28 (43.80)	
Minimal consumption	58 (50.40)	28 (54.90)	30 (46.90)	
Usual consumption	8 (7.00)	2 (3.90)	6 (9.40)	
*Smoking habit, n (%)*				0.294
Never smoked	81 (70.40)	39 (76.50)	42 (65.60)	
Quit smoking	25 (21.70)	10 (19.60)	15 (23.40)	
Smoker	9 (7.80)	2 (3.90)	7 (10.90)	
*Education level, n (%)*				.813
Read and write	39 (33.90)	15 (29.40)	24 (37.50)	
Elementary, intermediate	48 (41.70)	22 (43.10)	26 (40.60)	
Secondary, vocational	24 (20.90)	12 (23.50)	12 (18.80)	
University	4 (3.50)	2 (3.90)	2 (3.10)	

TKA, total knee arthroplasty; UKA, unicompartmental knee arthroplasty

The longitudinal analysis of pain trajectory variables revealed a statistically significant temporal reduction in pain intensity across all three evaluated aspects (resting, walking, and knee bending) in both the TKA and UKA cohorts (P < 0.001). No significant intergroup differences were detected at any measurement time point for these pain variables. Similarly, the functional trajectory variables exhibited significant improvements over time in measures of active knee flexion (P < 0.001), walking speed (P < 0.001), physical performance (P < 0.001), and overall health functioning (P < 0.001) within both groups. Notably, between-group analyses demonstrated a significantly greater ROM at one-week post-surgery in the UKA group (P = 0.034), as well as a significantly improved walking speed at the same time point (P = 0.011). Regarding psychological trajectory variables, the TKA group presented significant reductions over time in pain catastrophizing (P = 0.025), fear of movement related to pain (P = 0.007), and depressive symptoms (P = 0.046). In the UKA group, significant temporal decreases were observed in pain catastrophizing (P < 0.001) and anxiety symptoms (P = 0.017). A between-group analysis at one-week post-surgery identified a significantly higher level of pain-related fear of movement (p = 0.007) in the TKA group compared to the UKA group. These findings suggest distinct temporal patterns in the evolution of pain, functional, and psychological outcomes post-knee arthroplasty, with some differences observed in the acute phase between TKA and UKA groups in specific outcome measures (Table 2).

**Table 2:** Postoperative pain, functional and psychological trajectories between TKA and UKA subjects

Variable	TKA subjects (N = 51)						UKA subjects (N = 64)						Between-group comparison
	1 week^a^	4 weeks^b^	12 weeks^c^	24 weeks^d^	F	p-value	1 week^a^	4 weeks^b^	12 weeks^c^	24 weeks^d^	F	p-value	
Pain at rest (0–10)	4.46 (2.47)	3.45 (2.35)	2.09 (2.15)	1.80 (2.18)	29.57	< 0.001	3.87 (2.61)	2.94 (2.51)	2.45 (2.44)	1.65 (2.29)	24.31	< 0.001	0.284^a^, 0.213^b^, 0.597^c^, 0.442^c^
Pain while walking (0–10)	5.17 (2.52)	4.19 (2.80)	2.97 (2.60)	2.69 (2.97)	14.77	< 0.001	4.30 (2.56)	3.80 (2.44)	3.45 (2.58)	2.84 (2.42)	7.74	< 0.001	0.076^a^, 0.504^b^, 0.312^c^, 0.387^d^
Pain during knee flexion (0–10)	6.71 (2.33)	5.62 (2.61)	3.76 (2.50)	2.93 (2.74)	31.06	< 0.001	6.23 (2.37)	5.30 (2.60)	4.47 (2.84)	3.43 (2.63)	26.84	< 0.001	0.308^a^, 0.586^b^, 0.165^c^, 0.269^d^
Active knee flexion (degrees)	76.67 (15.67)	98.30 (13.80)	108.54 (11.79)	109.86 (9.89)	67.83	< 0.001	83.44 (16.66)	99.29 (20.04)	108.85 (13.85)	114.25 (14.32)	51.37	< 0.001	0.034^a^, 0.229^b^, 0.421^c^, 0.069^d^
4 m walking test (m/s)	0.46 (0.22)	0.76 (0.24)	1.03 (0.29)	1.03 (0.34)	67.37	< 0.001	0.58 (0.26)	0.85 (0.29)	1.01 (0.30)	1.09 (0.34)	70.93	< 0.001	0.011^a^, 0.077^b^, 0.905^c^, 0.345^d^
30 s Chair stand test (repetitions)	3.27 (3.79)	7.96 (4.26)	11.05 (3.65)	10.78 (4.70)	68.67	< 0.001	4.48 (4.23)	7.77 (4.31)	9.71 (4.14)	10.38 (4.82)	63.54	< 0.001	0.100^a^, 0.953^b^, 0.299^c^, 0.873^d^
WOMAC (0–96)	44.12 (17.51)	29.13 (14.16)	20.08 (16.71)	17.77 (16.53)	44.27	< 0.001	40.31 (19.53)	27.98 (16.81)	21.97 (16.02)	17.48 (14.18)	49.16	< 0.001	0.231^a^, 0.302^b^, 0.467^c^, 0.658^d^
Pain catastrophizing (0–52)	19.08 (14.94)	17.21 (13.43)	14.80 (14.67)	12.86 (15.17)	3.46	.025	17.42 (12.91)	13.61 (11.31)	10.77 (10.56)	10.76 (11.54)	14.50	< 0.001	0.679^a^, 0.210^b^, 0.297^c^, 0.974^d^
Pain-related fear of movement (11–44)	31.04 (4.85)	29.11 (5.62)	28.39 (6.26)	28.46 (7.58)	4.64	.007	28.13 (5.86)	28.39 (6.55)	28.13 (6.42)	27.59 (5.91)	0.33	.801	0.007^a^, 0.639^b^, 0.828^c^, 0.824^d^
Anxiety symptoms (0–14)	6.59 (4.70)	5.36 (4.72)	5.34 (5.34)	5.03 (5.53)	1.38	.264	5.97 (5.14)	5.53 (4.83)	4.65 (4.50)	4.61 (4.90)	3.71	.017	0.280^a^, 0.773^b^, 0.893^c^, 0.990^d^
Depressive symptoms (0–14)	4.90 (3.84)	3.74 (3.52)	4.02 (4.25)	4.92 (4.99)	2.91	0.46	4.69 (4.11)	4.74 (4.39)	3.94 (4.27)	4.48 (4.73)	1.69	0.179	649^a^, 0.235^b^, 0.968^c^, .754^d^

TKA, total knee arthroplasty; UKA, unicompartmental knee arthroplasty; 4 m, 4-m; 30 s, 30-s; WOMAC, Western Ontario and McMaster Universities Arthritis Index

^a^between-group comparison 1 week after surgery

^b^Between-group comparison 4 weeks after surgery

^c^Between-group comparison 12 weeks after surgery

^d^Between-group comparison 24 weeks after surgery

The analysis targeting potential controlling variables only underscored male sex (P = 0.026) and lower body mass index (BMI) (P < 0.001) as significant determinants positively influencing the evolution of walking speed trajectories over time. Accordingly, these variables were incorporated as controls in further model analyses. Finally, the inclusion of surgery type as a variable in various models revealed no significant impact on the trajectories of pain, functional, and psychological outcomes throughout the six-month rehabilitation period (Table 3).

**Table 3:** Type of surgery (TKA or UKA) effect on pain, functional and psychological trajectories

Dependent variable	Estimate (SE)	F	p-value	Lower bound	Higher bound
Pain during knee flexión	0.472 (0.513)	0.057	0.360	− 0.545	1.488
Pain while walking	0.090 (0.514)	0.264	0.861	− 0.928	1.108
Pain at rest	− 0.252 (0.428)	0.627	0.557	− 1.099	0.596
Active knee flexión	2.938 (2.751)	1.181	0.289	− 2.535	8.411
4 m Walking Test	0.079 (0.042)	3.495	0.064	− 0.005	0.162
30 s chair stand test	− 0.381 (0.907)	0.013	0.675	− 2.178	1.415
WOMAC	− 1.402 (2.946)	1.047	0.625	− 7.242	4.438
Pain catastrophizing	− 3.166 (2.591)	2.316	0.224	− 8.303	1.971
Pain-related fear of movement	− 0.850 (1.315)	1.597	0.519	− 3.456	1.757
Anxiety symptoms	− 0.986 (1.005)	0.730	0.329	− 2.978	1.006
Depressive symptoms	− 0.999 (0.950)	0.157	0.296	− 2.882	0.885

TKA, total knee arthroplasty; UKA, unicompartmental knee arthroplasty; SE, standard error; 4 m, 4-m; 30 s, 30-s; WOMAC, Western Ontario and McMaster Universities Arthritis Index

## Discussion

The primary objective of this study was to ascertain the potential impact of surgery type on pain, functional, and psychological trajectories during a six-month rehabilitation period. The results suggest that, while specific acute phase differences exist between total knee arthroplasty and unicompartmental knee arthroplasty groups, the type of surgery does not significantly influence the overall rehabilitation trajectory.

UKA is often promoted for its advantages over TKA, including quicker recovery, fewer complications, and better functionality(25). Studies such as the meta-analysis by Wilson HA et al. (2019), have highlighted specific functional advantages for UKA over TKA, such as better range of motion, improved ability to kneel, and enhanced patient-reported outcomes(26). In the present study, however, UKA only showed significantly higher walking speed and range of motion in the early acute phase, with a near-significant trend favoring UKA in overall walking speed trajectory. These findings do not fully support the anticipated long-term benefits of UKA. Instead, they align with recent research suggesting that these differences are mainly present in the early phases, with similar clinical and functional outcomes observed between UKA and TKA as rehabilitation progresses. For example, a systematic review by Longo et al. (2015) found no significant differences in functional outcomes between the two procedures(27). Additionally, a recent meta-analysis reported that in follow-ups beyond 12 months, only 1 out of 8 studies showed better functional outcomes with UKA, while 3 of the 8 studies reported a higher range of motion with UKA compared to TKA(28). Given these conflicting findings on functional outcomes, other factors have been increasingly considered in clinical decision-making, such as operative time, hospital stay duration, complication rates, and rehabilitation needs(28). Future studies assessing these variables may provide orthopedic surgeons with more comprehensive data to inform their surgical choices.

The present study indicates that general clinical characteristics of participants do not significantly influence pain or functional outcomes for TKA or UKA, except of male sex and lower BMI, which seems to be correlated with better walking speed. This finding aligns with recent research showing no difference in pain and activity levels between TKA and UKA patients over a two-year postoperative period. However, male sex, younger age, and lower BMI appear to positively influence lower extremity activity levels (29). Specifically, regarding walking speed, female sex, older age, lower preoperative function, and higher BMI have been associated with significantly and clinically lower gait speed(30). These associations have predominantly been observed in studies with short to mid-term follow-ups and need further clarification in studies with longer follow-up periods. Additionally, the correlation between BMI and postoperative functional outcomes, such as range of motion or walking speed, does not appear to be influenced by BMI one year after surgery. This suggests that the impact of BMI on these outcomes may diminish over time with longer follow-up assessments(31).

Preoperative patient education is as an essential component of clinical pathways for lower limb arthroplasty(32). This process should consider patients' expectations, including psychological and organizational preparation(33). The findings of the present study provide evidence-based insights to enhance patient education by offering detailed information about the rehabilitation process following UKA and TKA. Studies indicate that mismatched expectations between patients and surgeons can lead to increased dissatisfaction(10,34,35). Additionally, previous research suggests that patients' distrust in medical procedures could be a significant predictor of postsurgical chronic pain development(24). Patients should be informed that there are no differences in pain, functional, or psychological trajectories between the two surgical procedures. However, TKA patients may experience higher levels of pain-related fear of movement than UKA patients. It has been observed that TKA patients tend to have a higher awareness of their artificial joint(36), even though both interventions are expected to have similar overall outcomes(28). Given that fear of movement can impact postoperative recovery(37), patient education should emphasize reducing this fear. Effective educational strategies include pain neuroscience education, which involves explaining the processes underlying inflammation, nociception, and pain perception(38). This approach helps patients understand their postoperative symptoms better, thereby reducing hypervigilance, rumination, and activity avoidance due to fear of movement(39).

This study has several limitations that should be acknowledged. One potential limitation is selection bias, as the sample was initially chosen to address a different hypothesis, which may not be representative of the general population. Additionally, the study may lack statistical power due to the absence of a specific sample size calculation for this secondary analysis, potentially limiting the ability to detect significant differences or associations. There is also a risk of confounding bias, as the original study design may not have accounted for all variables relevant to this secondary analysis. Another limitation is the short follow-up period, which may not capture long-term outcomes such as implant durability, late complications, or the need for revision surgery. A longer follow-up is necessary to fully assess these aspects and provide a more comprehensive evaluation of both procedures. Furthermore, results on ROM should be interpreted carefully. Range of motion can vary depending on the TKA design, whether posterior-stabilized or cruciate-retaining, and information on the type of TKA implanted was not available. Additionally, preoperative ROM data was missing, which means the range of motion prior to surgery could not be assessed. Finally, the study is constrained by the design of the original study, which may impose methodological limitations affecting the internal and external validity of the secondary analysis. Thus, the results should therefore be interpreted with caution, as the study was not specifically designed to answer the new research question.

## Conclusion

Despite differences in the acute phase regarding range of motion, walking speed, and pain-related fear of movement, there are no differences in pain, functional, and psychological trajectories throughout the six-month rehabilitation period between UKAs and TKA. These results should be acknowledged to better inform patients before deciding the type of intervention and to improve patient education during the perioperative period.

## Data Availability

The data derived from this study that support the findings are available upon reasonable request from the corresponding author.
